# Supplementary material for: Causal Relationship between Aging and Anorexia Nervosa: A White-Matter-Microstructure-Mediated Mendelian Randomization Analysis
Source: Biomedicines. 2024 Aug 16;12(8):1874. doi: 10.3390/biomedicines12081874 (PMC11351342; doi:10.3390/biomedicines12081874)
Supplement: Supplementary file 1 [file biomedicines-12-01874-s001.zip › Supplementary Figure/Figure S8/Leaveoneout plot of MR analysis for TL to ubm-b-2013.pdf]

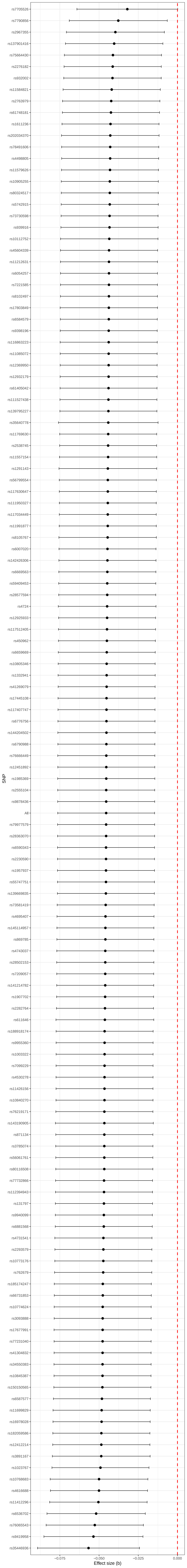

rs7705526
rs7790856
rs2967355
rs137901416
rs75664430
rs2276182
rs932002
rs11584821
rs2763979
rs61748181
rs1611236
rs202034370
rs78491606
rs4498805
rs11579626
rs10905255
rs80324517
rs5742915
rs73730598
rs939916
rs10112752
rs45604339
rs11212631
rs6054257
rs7221585
rs8102497
rs17803849
rs6584579
rs9398196
rs116863223
rs11085072
rs12369950
rs12932179
rs61405042
rs111527438
rs139795227
rs35640778
rs11769630
rs2538745
rs11557154
rs1291143
rs56799554
rs117630647
rs111950327
rs117034449
rs11991877
rs8105767
rs6007020
rs142426306
rs6669563
rs59409453
rs28577594
rs4724
rs12925933
rs117512405
rs450962
rs6659669
rs10805346
rs1332941
rs41269079
rs17445108
rs117407747
rs6776756
rs144204502
rs6790988
rs76666449
rs12451892
rs1985369
rs2555104
rs9878436
All
rs79977579
rs28363070
rs6590343
rs2230590
rs1957937
rs55747751
rs139669835
rs73581419
rs4695407
rs145114957
rs869785
rs4743037
rs28502153
rs7209057
rs141214782
rs1907702
rs2282764
rs611646
rs188918174
rs9955360
rs1003322
rs7099229
rs4530278
rs11426156
rs10840270
rs76219171
rs143190905
rs871134
rs3785074
rs56061761
rs80116508
rs77732866
rs112394943
rs131797
rs9940099
rs6881568
rs4731541
rs2293579
rs10773176
rs762679
rs185174247
rs66731853
rs10774624
rs3093888
rs17677991
rs77231040
rs41304832
rs34550383
rs10845387
rs150150565
rs6587577
rs11699829
rs16978028
rs182059586
rs12412214
rs3891167
rs1023767
rs10768683
rs4616688
rs11412296
rs6536702
rs76065543
rs9419958
rs35446936
SNP
−0.075
−0.050
−0.025
0.000
Effect size (b)
